# The value of mentorship in fostering scientific potential among undergraduate dental students

**DOI:** 10.21142/2523-2754-1302-2025-236

**Published:** 2025-05-16

**Authors:** Angela Quispe-Salcedo

**Affiliations:** 1 Division of Anatomy and Cell Biology of the Hard Tissue, Niigata University Graduate School of Medical and Dental Sciences. Niigata, Japan. aquispesa@dent.niigata-u.ac.jp Niigata University Division of Anatomy and Cell Biology of the Hard Tissue Niigata University Graduate School of Medical and Dental Sciences Niigata Japan aquispesa@dent.niigata-u.ac.jp

Many of us probably associate the terms “mentoring” or “mentorship” with the traditional teacher-student relationship that we experienced at school, or perhaps, to the relationship “boss-employee” at any organization. In any event, the term “mentoring” is generally acknowledged as the involvement of a person with more experience (the mentor), and a less experienced one (the mentee) that needs to be advised in specific areas.

Mentoring relationships focus on helping the mentee’s growth and the accomplishment of his/her goals, including several approaches to doing so^1^^,^^2^. A more precise definition can describe mentoring as person-centered, long-term transformational process that requires the establishment of a trustful, respectful and confidential relationship between the mentor and mentee^3^. Importantly, this process may provide for mentee -and also mentor- psychosocial (psychological support and role modeling) and career support, in terms of career guidance, skill development, and sponsorship^2^. Therefore, the establishment of such relationship in early years of professional training can be determinant to find a better career route aligned to the true academic interests of the student, instead of the “conventional” career path that is chosen by default due to unawareness of the alternative options available.

It is the mission of every dental school all over the world to give their students the best education possible in clinical and scientific settings, so they may become competent dentists and contribute to the improvement of oral health. However, there is a small number of students that, along the progression of their dental education, may not feel interested in becoming clinical dentists at a full time, at least. These students may have strong academic interests in areas as diverse as oral epidemiology, anatomopathological sciences, dental basic sciences, forensics, and anthropological dental research. In fact, in countries with less developed scientific schemes, these fields are not subject of research interest and/or funding, leading to a negative circle for students, who usually give up on their very particular scientific interests and turned out into dentists with poor motivation for clinical work. 

However, in recent years, the new generation of dentists has begun to explore these less-explored areas, and after graduation of their doctoral programs, they begin to work in research laboratories located in countries with greater opportunities and funding for research of all kinds. As educators it is important to be open to listening the interests of our undergraduate students, who may reach us for career advice. It is crucial to provide them with some kind of orientation and make an effort to link them with colleagues who are active in these areas rather than closing off their alternatives for a career in less-known disciplines within dental sciences. It is therefore important that new generations of researchers, who were able to go abroad to develop their research potential, extend support to those who need it.

We should not forget that there are undergraduate dental students who wish to explore beyond clinical dentistry, with all the motivation to become great epidemiologists or specialists in tooth development and morphogenesis, for example. In countries where research is not yet fully established, the creation of mentoring programs would be a potent way of achieving career development and furthering the scientific advancement of our profession. 
